# Three new species of the genus *Centistidea* Rohwer, 1914 (Hymenoptera, Braconidae, Miracinae) from India and Saudi Arabia

**DOI:** 10.3897/zookeys.889.34942

**Published:** 2019-11-14

**Authors:** Hamed A. Ghramh, Zubair Ahmad, Kavita Pandey

**Affiliations:** 1 Research Center for Advanced Materials Science (RCAMS), King Khalid University, P.O. Box 9004, Abha 61413, Saudi Arabia; 2 Unit of Bee Research and Honey Production, Faculty of Science, King Khalid University, P.O. Box 9004, Abha 61413, Saudi Arabia; 3 Biology Department, Faculty of Science, King Khalid University, P.O. Box 9004, Abha 61413, Saudi Arabia; 4 Biology Department, Faculty of Sciences and Arts, Dhahran Al Jounub, King Khalid University, Saudi Arabia; 5 Department of Zoology, Aligarh Muslim University, Aligarh, 202002, UP India

**Keywords:** *Acrocercops phaeospora*, *Cosmopteryx phaeogastra*, India, leafminer, parasitoids, *Phaseolus cylindrica*, Saudi Arabia, *Syzigium cuminii*

## Abstract

*Centistidea acrocercopsi* Ahmad & Pandey, **sp. nov.**, *C. cosmopteryxi* Ahmad & Pandey, **sp. nov.**, and *C. tihamica* Ghramh & Ahmad, **sp. nov.** are described as new to science. The genus *Centistidea* Rohwer (Hymenoptera: Braconidae: Miracinae) is recorded for the first time from Saudi Arabia. Two species were reared from *Acrocercops phaeospora* Meyrick and *Cosmopteryx phaeogastra* (Meyrick) in India, while *Centistidea tihamica* was collected by Malaise trap in Saudi Arabia. Characters of these new species and their affinities with related taxa are discussed. Data on habitat, host records, and host plant species for all the parasitoid species are also provided.

## Introduction

The subfamily Miracinae is a small cosmopolitan subfamily with two genera, *Centistidea* Rohwer, 1914 and *Mirax* Haliday, 1833, bearing 56 species worldwide (Papp 2013; Cauich-Kumul et al. 2014; Farahani et al. 2014; Yu et al. 2016; Ranjith et al. 2018). Members of this subfamily are solitary koinobiont endoparasitoids which usually attack leaf-mining caterpillars of lepidopteran families, viz., Nepticulidae, Tischeriidae, Heliozelidae, Lyonetiidae, and Gracillariidae (Maetô 1995; Memmott et al. 1994; Shaw and Huddleston 1991). This subfamily is characterized by the presence of a Y-shaped structure formed by the sclerotized part of the first three metasomal tergites, surrounded by membranous lateral parts, a reduced wing venation, the antenna being 14-segmented, and the compound eyes setose. The subfamily Miracinae has been studied by the following workers: Palaearctic Region (Papp 1984; Western Palaearctic, Tobias 1986; former USSR, Papp 1987; Korea, Belokobylskij 1989; East Palaearctic, Maetô 1995; Japan, Papp and Chou 1996; Taiwan, Wu et al. 2000; China, Beyarslan 2009; Turkey), Oriental Region (van Achterberg and Mehernejad 2002; North Oriental, Farahani et al. 2014; Iran, Ranjith et al. 2018; South India), and Neotropical Region (Papp 2013; Colombia and Honduras, Cauich-kumul et al. 2014; Mexico).

*Centistidea* Rohwer is a small genus with 27 described species worldwide (Yu et al. 2016; Ranjith et al. 2018). The genus *Centistidea* differs from *Mirax* in having the propodeum with medio-longitudinal carina and the notauli usually shallowly impressed anteriorly (van Achterberg and Mehernejad 2002; Papp 2013). Recently, Ranjith et al. (2018) described seven new species of *Centistidea* from the southern part of the Indian peninsula. In the present work, three new species of *Centistidea* are described as new to science, of which two are from the northern part of India and one species, *Centistidea tihamica* sp. nov., is described from Saudi Arabia.

## Materials and methods

The Indian specimens were collected from western Uttar Pradesh (north India) in order to identify the parasitoids of leaf miners along the roadside at Aligarh Muslim University campus. Saudi specimens were collected by Malaise trap from Tihama in Asir region (southwestern Saudi Arabia). We have followed van Achterberg (1988) for the terminology of various body parts and wing venation, and Eady (1968) for terminology of micro-sculpture. The specimens were deposited in the Insect Collection of the Department of Zoology, Aligarh Muslim University, Aligarh, India (**ZDAMU**).

## Taxonomic accounts

### Centistidea acrocercopsi

Taxon classificationAnimaliaHymenopteraBraconidae

Ahmad & Pandey sp. nov.

45344B8E-FEED-5437-8BEA-14243569C8B5

http://zoobank.org/58BABAEF-CDF0-4C96-90A9-37A3FAD2FA3E

Figs 1–4

#### Material examined.

**Holotype**: INDIA • ♀: Uttar Pradesh, Etah, 7.VIII.2004; ex. *Acrocercops phaeospora* (Meyrick) on *Syzygyium cuminii*, Z. Ahmad leg. (ZDAMU). **Paratype**: 1♀, with same data as holotype (HB-138, ZDAMU).

#### Diagnosis.

Following the key to East Palearctic and Oriental species of the genus *Centistidea* Rohwer (Ranjith et al. 2018), *C. acrocercopsi* sp. nov. keys near to *C. rugator* (Ranjith et al. 2018); however, it differs in the following characters (*C. rugator* in parentheses): (i) body largely yellowish (body largely yellowish except tergites 1–6 dark brown dorsally), (ii) length of eye 1.3 × long as temple in dorsal view (length of eye 2.90 × as long as temple), (iii) ovipositor sheath 0.10 × as long as fore wing length (0.20 × as long as forewing), (iv) first tergite smooth, widening medially, slightly narrowing basally and apically, 4.0 × as long as its maximum width (first tergite smooth, widening medially, distinctly narrowing basally and apically, 3.0 × as long as its maximum width).

#### Description.

Female: body length: 1.7 mm; length of fore wing: 2.1 mm; length of antenna: 1.7 mm.

***Head:*** ca. 2 × as wide as long in dorsal view; length of eye 1.3 × as long as temple in dorsal view; temple and vertex shiny with indistinct punctures; OOL: POL: AOL: OD = 6: 3: 2: 1.5; inner margin of eyes subparallel; face distinctly convex medially, flattened laterally and almost smooth and shiny; clypeus smooth and evenly convex; malar space ca. 2 × as long as basal width of mandible; antenna 14-segmented, F_1_ as long as F_2_, apical flagellomere pointed.

***Mesosoma:*** 1.4 × as long as wide; mesoscutum shiny with indistinct punctures, notauli only anteriorly impressed and finely crenulate; prescutellar furrow poorly developed without any groove; scutellum smooth and shiny, medio-posterior depression of scutellum elliptical; propodeum somewhat smooth, with a complete median longitudinal carinae and two transverse carinae posteriorly, few rugosities adjacent to median longitudinal carina; meso- and metapleuron almost smooth and shiny.

***Wings:*** Pterostigma with long and slender apical expansion, 2.7 × longer than wide, vein r issuing from its middle; vein 1-M 1.5 × longer than vein m-cu; vein 1-CU1 0.9 × as long as vein 2-CU1.

***Legs:*** Hind coxa smooth, length of femur, tibia, and basitarsus of hind leg 3.0 ×, 7.1 ×, and 4.0 × their maximum width, respectively; length of hind tibial spur 0.30 × as long as hind basitarsus. Hind tarsal claw large and without acute lobe.

***Metasoma:*** 1.5 × as long as wide; first tergite, smooth, widening medially, slightly narrowing basally and apically, 4.0 × as long as its maximum width; second tergite sclerotized with strong longitudinal striations; hypopygium small, membranous, desclerotized, sparsely setose at apex, not surpassing end of metasoma; ovipositor thick, setose, distinctly shorter than petiole and hind basitarsus.

***Color:*** Largely yellow except for the following: antenna (except for scapus and pedicel yellow), veins, pterostigma, and ovipositor apically brown; wings moderately infuscate apically.

**Figures 1–4.**
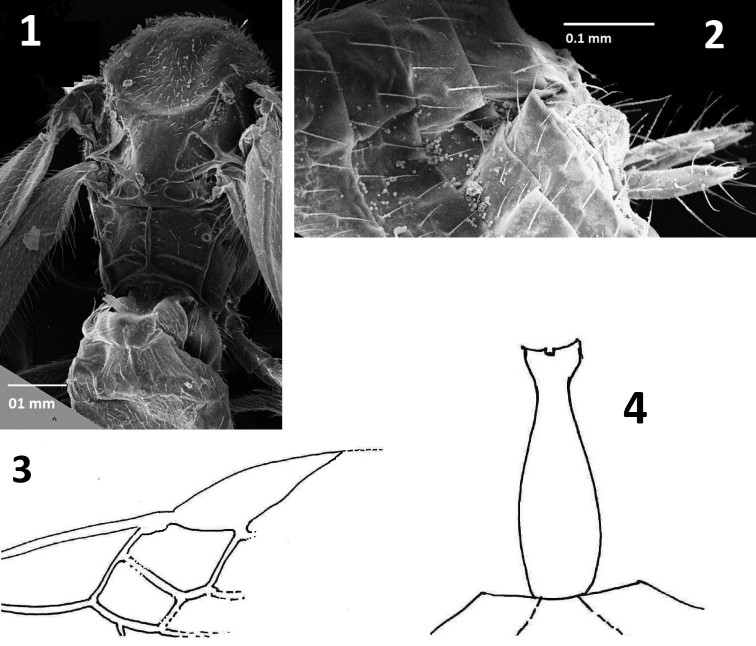
*Centistidea acrocercopsi* sp. nov. **1** mesosoma and metasoma, dorsal view **2** hypopygium **3** forewing **4** metasomal T1 and T2.

#### Male.

Unknown.

#### Host.

*Acrocercops phaeospora* (Meyrick).

#### Distribution.

India: Uttar Pradesh.

#### Etymology.

The new species is named after its host insect.

### Centistidea cosmopteryxi

Taxon classificationAnimaliaHymenopteraBraconidae

Ahmad & Pandey sp. nov.

37DB0B72-3E78-5892-A74C-CEF1FF50E8D3

http://zoobank.org/BF7CFA22-06E7-4308-A0F6-D839996B4013

Figs 5–10

#### Material examined.

**Holotype**: INDIA • ♀; Uttar Pradesh, Etah, 5.x.2004; ex. *Cosmopteryx phaeogastra* (Meyr) on *Phaseolus cylindrica* (coll. Z Ahmad) (ZDAMU). **Paratype**: 1♀, with same data as holotype (HB-139, ZDAMU).

#### Diagnosis.

Following the key to East Palaearctic and Oriental species of the genus *Centistidea* Rohwer (van Achterberg and Mehernejad 2002; Ranjith et al. 2018), *C. cosmopteryxi* sp. nov. keys with *C. sii* (Maetô, 1995) on the presence of yellowish head, notauli only anteriorly impressed and finely crenulate, and vein 1-R1 of fore wing distinctly vein-like. This combination of characters is quite unique among the genus *Centistedea*. However, the new species differs in the following characters: (i) wings slightly infuscate (wings hyaline in *C. sii*), (ii) length of eye 1.8 × temple (dorsal length of eye 1.10 × temple in *C. sii*), (iii) length of first tergites 2.3 × its maximum width and 3.2 × its apical width (length of first tergites 3–3.5 × its maximum width and 3.2 × its apical width in *C. sii*). When considering the similarities of characters like vein 1-CU1 of fore wing 0.9 × as long as vein 2-CU1 and scutellum with oval pits medio-posteriorly, then the new species runs near to *C. mogra* (Papp 1987). However, it differs in the following characters: (i) sub-alar depression of fore wings finely aciculate (sub-alar depression of fore wings smooth in *C. mogra*), (ii) propodeum with some rugosity on anterior part of median longitudinal carina (propodeum without any rugosity on anterior part of median longitudinal carina in *C. mogra*), (iii) vein 1-CU1 slightly shorter than 2-CU1 (vein 1-CU1 of fore wing as long as 2-CU1 in *C. mogra*), and (iv) mesonotum complete dark brown (mesonotum tinged with brown in *C. mogra*).

#### Description.

**Holotype**: Female body length: 2.0 mm; length of forewing: 2.1 mm; length of antenna: 2.0 mm.

***Head:*** 1.9 × as wide as long in dorsal view (12 : 23); length of eye 1.8 × temple (9 : 5) in dorsal view: head and vertex indistinctly punctate; OOL; POL : AOL: OD = 4: 2: 1: 2; inner margin of eyes subparallel; face distinctly convex medially, flattened laterally smooth; clypeus smooth and evenly convex; malar space 0.9 × as long as basal width of the mandible; antennae with 14 segments, F_1_ ca. 5 × as long as wide, 1.1 times longer than F_2_, penultimate flagellomere 2.5–3.0 × as long as wide and apical flagellomere pointed.

***Mesosoma:*** 1.5 × as long as wide; mesoscutum shiny with few distinct punctures, notauli only anteriorly impressed and finely crenulate; prescutellar furrow distinct as a narrow groove with few crenulations; scutellum almost smooth and shiny, medio-posterior depression of scutellum oval and moderately close to each other; propodeum almost smooth (except few rugosity on anterior part of median longitudinal carina) with a complete median longitudinal carina bifurcate posteriorly near the end of propodeum, median carina of propodeum absent behind level of costulae; pair of membranous white spots at side of pronotum distinct, mesopleuron and metapleuron smooth.

***Wings:*** Pterostigma with a long slender, apical expansion, 2.4 × longer than wide; vein 1-R1 of fore wing distinctly vein-like; vein r issuing from its middle; vein 1-M 1.6 × longer than vein m-cu; vein 1-CU1 of fore wing 0.9 × as long as vein 2-CU1.

***Legs:*** Hind coxa smooth, lengths of hind femur, tibia, and basitarsus of hind leg 3.0, 7.0, and 4.5 × their maximum widths, respectively; length of hind tibial spurs 0.26 × and 0.33 × as long as hind basitarsus.

***Metasoma:*** Ca. 2.0 × as long as wide; first tergite smooth, widening medially, distinctly narrowing basally and apically, 3.2 × as long as its maximum width; T_2_ subtriangular, smooth, laterally membranous and longitudinally striated; T_3_ longitudinally striated; ovipositor sheaths setose at apical half 0.1 × as long as forewing; hypopygium smooth medially folded, truncate apically, weakly sclerotized and setose.

***Color:*** Yellowish brown except for the following: antennae, mesonotum, and metasoma dark brown to blackish brown; propleuron, mesopleuron, metapleuron, and ovipositor brown; T_3_, laterotergites yellow; wings infuscate.

**Figures 5–10.**
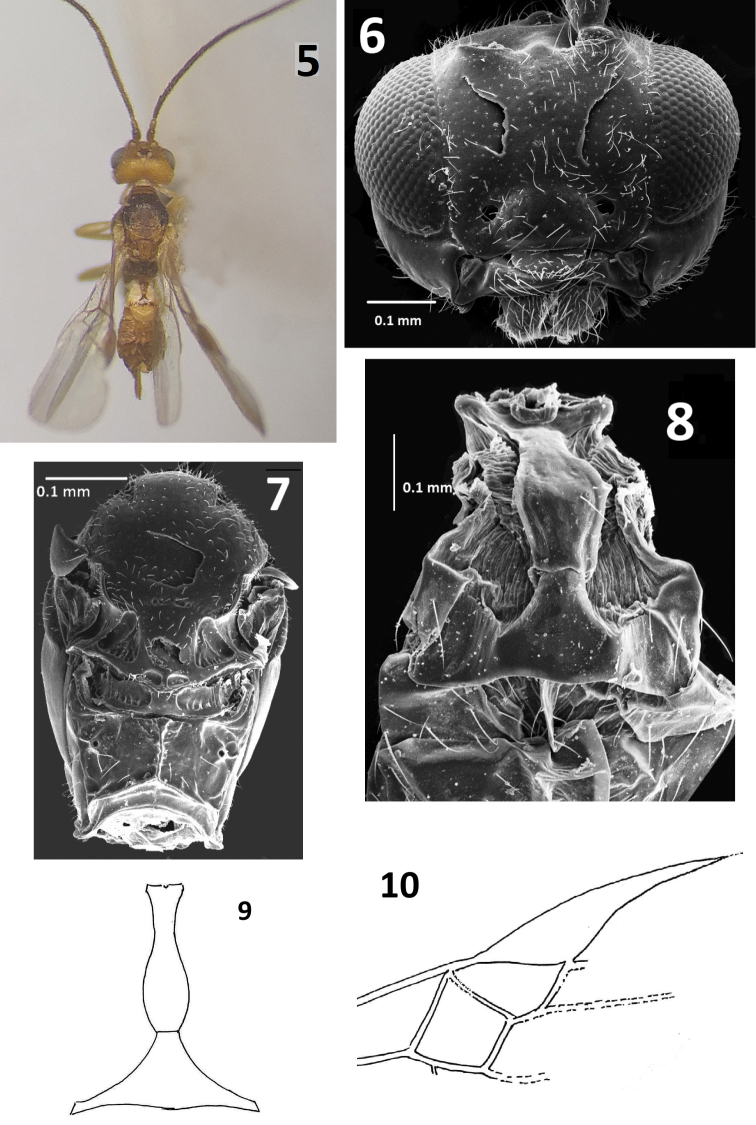
*Centistidea cosmopteryxi* sp. nov. **5** body profile, dorsal view **6** head, frontal view **7** mesosoma, dorsal view **8** metasoma, dorsal view **9** metasomal T1 and T2 **10** forewing.

#### Male.

Unknown.

#### Host.

*Cosmopteryx phaeogastra* (Meyrick).

#### Distribution.

India: Uttar Pradesh.

#### Etymology.

The new species is named after its host insect.

### Centistidea tihamica

Taxon classificationAnimaliaHymenopteraBraconidae

Ghramh & Ahmad sp. nov.

E28F67CA-1450-5E75-A439-3152750ADDB3

http://zoobank.org/4B0EEBD0-E096-4325-9089-AB0C0B931574

Figs 11–13

#### Material examined.

**Holotype**: Saudi Arabia • ♀; Abha, Tihama, 5.x.2015; malaise trap (coll. Z Ahmad). **Paratype**: 3 ♀; with same data as holotype (HB-139, ZDAMU).

#### Diagnosis.

Following the key to East Palaearctic and Oriental species of the genus *Centistidea* Rohwer (Ranjith et al. 2018), *C. tihamica* sp. nov. keys near *C. zhaoi* Chen et al., 1997; however, it differs in the following characters: (i) mesoscutum blackish brown (mesoscutum yellowish), (ii) dorsal length of eye 1.5 × temple (dorsal length of eye 1.80 × temple), (iii) first flagellomere 1.25 × as long as second flagellomere (first flagellomere 1.30 × as long as second flagellomere), (iv) mesonotum complete dark brown (mesonotum tinged with brown).

#### Description.

**Holotype**: Female: body length: 1.8 mm; length of forewing: 1.9 mm; length of antenna: 1.8 mm.

***Head:*** 2.0 × as wide as long in dorsal view, distinctly wider than the mesosoma dorsally; length of eye 1.5 × temple in dorsal view: head and vertex indistinctly punctate; OOL: POL : AOL: OD = 4: 2: 1: 2; inner margin of eyes subparallel; face distinctly convex medially, flattened laterally smooth; clypeus smooth and evenly convex; malar space 0.9 × as long as basal width of the mandible; antenna with 14 segments, F_1_ ca. 6 × as long as wide, 1.25 × longer than F_2_, penultimate flagellomere 2.5–3.0 × as long as wide, and apical flagellomere pointed.

***Mesosoma:*** 1.5 × as long as wide; mesoscutum shiny with few distinct punctures, notauli only anteriorly impressed; prescutellar furrow distinct, present as a narrow groove and crenulations; scutellum almost smooth and shiny, medio-posterior depression of scutellum semicircular; propodeum almost smooth with a complete median longitudinal carina bifurcate posteriorly, median carina of propodeum absent behind level of costulae, posterior part clearly differentiated from dorsal part of propodeum; mesopleuron and metapleuron smooth.

***Wings:*** Pterostigma with a long slender, apical expansion, 2.2 × longer than wide; vein r very prominent and 0.2 × as long as the height of pterostigma, vein 1-M 1.5 × longer than vein m-cu; vein 1-CU1 of fore wing 0.7 × as long as vein 2-CU1.

***Legs:*** Hind coxa smooth, lengths of hind femur, tibia, and basitarsus of hind leg 3.0, 7.0, and 4.0 × their maximum widths, respectively; length of hind tibial spurs 0.23 × and 0.32 × as long as hind basitarsus.

***Metasoma:*** Ca. 2.0 × as long as wide; first tergite, smooth, widening medially, distinctly narrowing basally and apically, 4.0 × as long as its maximum width; T_2_ subtriangular, smooth, laterally membranous, and longitudinally striated; T_3_ longitudinally striated; ovipositor sheaths 0.15 × as long as forewing; hypopygium smooth, medially folded, truncate apically, weakly sclerotized, and setose.

***Color:*** Yellowish brown except for the following: head and legs yellowish; antennae, mesosoma, and metasoma dark brown to blackish brown; laterotergites yellowish; wings slightly infuscate.

**Figures 11–13.**
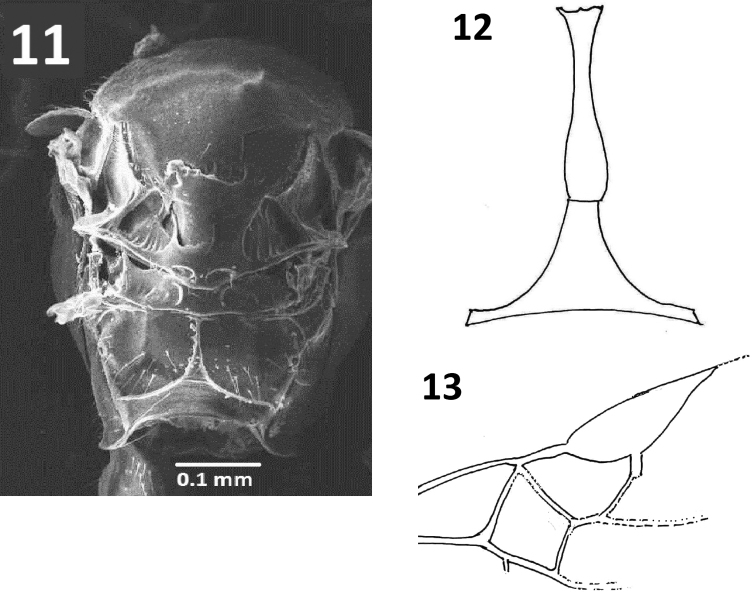
*Centistidea tihamica* sp. nov. **11** mesosoma, dorsal view **12** metasomal T1 and T2 **13** forewing.

#### Male.

Unknown.

#### Host.

Unknown.

#### Distribution.

Saudi Arabia: Abha.

#### Etymology.

The new species is named after its locality.

## Discussion

In this study, *Centistidea tihamica* sp. nov. is described from the southwestern region of Saudi Arabia, thus increasing the distributional range of the genus *Centistidea* to the Afrotropical region. The genus *Centistidea* is very well represented in almost all zoogeographical regions except for Northwestern Palearctic and the Afrotropical region (van Achterberg and Mehernejad 2002; Yu et al. 2016; Ranjith et al. 2018). Southwest Saudi Arabia is divided by steep rocky mountains into two main subdivisions, a lowland coastal plain at the west, known as “Tihama”, and a mountainous area with an elevation of 3,000 m highlands at its peak at the east, known as “Asir Mountains range (Alahmed et al. 2010; Ibrahim and Abdoon 2005). Although the geographical location of the southwestern region of Saudi Arabia is debatable, many workers have considered it to belong to the Afrotropical region (Sclater 1858; Wallace 1876 and Hölzel 1998). Studies of several taxonomic groups of insects have revealed that this region has a clear faunal similarity with the Afrotropical region (Cowie 1989; Mahnert et al. 2014; Sharaf et al. 2014; El-Hawagry and Al Dhafer 2015; Abdel-Dayem et al. 2018). In the present study two species, *Centistidea acrocercopsi* sp. nov. and *Centistidea cosmopteryxi* sp. nov., also extended the distribution of *Centistidea* to the northern part of India, as it was previously reported only from the southern part of the Indian Peninsula (Ranjith et al. 2018).

## Supplementary Material

XML Treatment for Centistidea acrocercopsi

XML Treatment for Centistidea cosmopteryxi

XML Treatment for Centistidea tihamica
